# *Bifidobacteriumlongum* subsp. *infantis* CECT7210 (*B. infantis* IM-1^®^) Displays In Vitro Activity against Some Intestinal Pathogens

**DOI:** 10.3390/nu12113259

**Published:** 2020-10-24

**Authors:** Lorena Ruiz, Ana Belén Flórez, Borja Sánchez, José Antonio Moreno-Muñoz, Maria Rodriguez-Palmero, Jesús Jiménez, Clara G. de los Reyes Gavilán, Miguel Gueimonde, Patricia Ruas-Madiedo, Abelardo Margolles

**Affiliations:** 1 Instituto de Productos Lácteos de Asturias (CSIC), P. Río Linares s/n, 33300 Villaviciosa, Spain; lorena.ruiz@ipla.csic.es (L.R.); abflorez@ipla.csic.es (A.B.F.); borja.sanchez@csic.es (B.S.); greyes_gavilan@ipla.csic.es (C.G.d.l.R.G.); mgueimonde@ipla.csic.es (M.G.); ruas-madiedo@ipla.csic.es (P.R.-M.); 2 Instituto de Investigación Sanitaria del Principado de Asturias (ISPA), 33011 Oviedo, Spain; 3 Laboratorios Ordesa S.L., Parc Científic de Barcelona, C/Baldiri Reixac 15-21, 08028 Barcelona, Spain; Josea.moreno@ordesa.es (J.A.M.-M.); mariarps@msn.com (M.R.-P.); Jesus.Jimenez@ordesa.es (J.J.)

**Keywords:** probiotics, prebiotics, synbiotic, *Bifidobacterium longum*, enteropathogens

## Abstract

Certain non-digestible oligosaccharides (NDO) are specifically fermented by bifidobacteria along the human gastrointestinal tract, selectively favoring their growth and the production of health-promoting metabolites. In the present study, the ability of the probiotic strain *Bifidobacterium longum* subsp. *infantis* CECT7210 (herein referred to as *B. infantis* IM-1^®^) to utilize a large range of oligosaccharides, or a mixture of oligosaccharides, was investigated. The strain was able to utilize all prebiotics screened. However, galactooligosaccharides (GOS), and GOS-containing mixtures, effectively increased its growth to a higher extent than the other prebiotics. The best synbiotic combination was used to examine the antimicrobial activity against *Escherichia coli*, *Cronobacter sakazakii*, *Listeria monocytogenes* and *Clostridium difficile* in co-culture experiments. *C. difficile* was inhibited by the synbiotic, but it failed to inhibit *E. coli*. Moreover, *Cr. sakazakii* growth decreased during co-culture with *B. infantis* IM-1^®^. Furthermore, adhesion experiments using the intestinal cell line HT29 showed that the strain IM-1^®^ was able to displace some pathogens from the enterocyte layer, especially *Cr. sakazakii* and *Salmonella enterica*, and prevented the adhesion of *Cr. sakazakii* and *Shigella sonnei*. In conclusion, a new synbiotic (probiotic strain *B. infantis* IM-1^®^ and GOS) appears to be a potential effective supplement for maintaining infant health. However, further studies are needed to go more deeply into the mechanisms that allow *B.* *infantis* IM-1^®^ to compete with enteropathogens.

## 1. Introduction

The gastrointestinal microbiota is a complex and dynamic ecosystem that inhabits the human gut from birth, and has an important influence on human health. The gut microbiota and the mucosa themselves act as barriers against invasion by potential pathogens, promoting normal intestinal function [1,2]. The indigenous microbiota prevents bacterial colonization by competing for the adhesion to the epithelium, producing specific antimicrobial compounds such as bacteriocins, and metabolizing specific nutrients towards short chain fatty acid (SCFA) and organic acids to create a restrictive environment, which is generally unfavorable for the growth of many enteric pathogens [3,4]. For this reason, nowadays, an increasing interest in developing functional foods and dietary supplements capable of promoting human health through beneficially modulating the gut ecosystem exists.

Bifidobacteria taxa are predominant in the large bowel (10^9^–10^11^ CFU/g feces), and they could represent from 3% to 7% of gut microbiota in adults [5,6,7], or even 91% in breastfed babies [8,9,10]. The well-documented health-promoting effects of the intestinal microbiota present in breastfed infants have prompted investigation into dietary approaches capable of establishing a similar microbiota structure, dominated by bifidobacteria, in formula-fed infants [11]. Indeed, it is currently accepted that nutrition and gut microbiota balancing in early life can significantly impact the immune programming development, conditioning health outcomes and the risk of suffering from chronic and inflammatory diseases in the short and long term, as recently reviewed [12]. The administration of probiotics, prebiotics, or synbiotics, which combine probiotics and prebiotics in the same formulation, are some of the dietary strategies capable to program the infant gut microbiota. Probiotics are live microorganisms that, when administered in adequate amounts, confer a health benefit on the host [13], while prebiotics are substrates that are selectively utilized by host microorganisms, conferring a health benefit [14]. Among the substrates meeting the prebiotic definition criteria, non-digestible carbohydrates have been the most studied to date. These include a wide array of oligo- and polysaccharides, such as inulin, fructooligosaccharides (FOS), galactooligosaccharides/transgalactosylatedoligosaccharides (GOS/TOS), xylooligosaccharides (XOS), arabinooligosaccharides (AOS), pectic oligosaccharides (POS) or lactulose-derived galactooligosaccharides (LDGOS), among others. Furthermore, these prebiotics can exert stimulatory effects on the immune system related to the production of some organic acids and increasing of intestinal beneficial bacteria, including bifidobacteria [15,16,17]. FOS and GOS fermentation lead to the production of acetate, butyrate and propionate, compounds that have been extensively investigated for their role in the maintenance of the host-homeostasis and health [18,19,20]. Regarding the use of probiotics, *Bifidobacterium* strains were found to be effective in inhibiting the growth of different enteropathogens, either through the production of inhibitor compounds, i.e., such as bacteriocins, or organic acids [21,22], through competition for nutrients [23], or through competition for adhesion sites, either using mucus or enterocyte adhesion models [24,25]. However, it has to be taken into account that health-promoting effects of a given probiotic are strain-specific. In addition, synbiotic formulations, including a mixture comprising live microorganisms and substrate(s) selectively utilized by host microorganisms that confers a health benefit on the host [26], can improve probiotic strains’ performance by enhancing their ability to colonize the human gut [27], or due to their effects on gut microbiota and immune modulation in early life [28].

*Bifidobacterium* strains originally isolated from breastfed infants have received great attention as potential probiotic strains for formula-fed infants in an attempt to act upon human components to produce beneficial functions/metabolites. Among these, the *B. longum* subsp. *infantis* CECT 7210 strain (herein referred to as *B. infantis* IM-1^®^), originally isolated from a breastfed infant feces, has previously been reported to confer protection against rotavirus infection both in animal and clinical trials [29,30,31,32], thus being an attractive probiotic strain for formula-fed infants. Indeed, whole genome comparison of *B. infantis* IM-1^®^ and *B. longum* subsp. *infantis* 157F [33], the most closely related strain, identified 340 extra genomic elements in the former that could be responsible for its protective effects against rotavirus [29], although additional analyses to determine the effect of strain 157F against rotavirus should be performed to corroborate the potential relationship between these lacking genes and the protective effects conferred by the strain IM-1^®^. In this context, the present work aimed at identifying a suitable synbiotic formula for infants feeding by testing the *B. infantis* IM-1^®^ strain with different combinations of prebiotic carbohydrates. The most attractive synbiotic combination was examined in co-cultures for their ability to inhibit the growth of enteropathogenic bacteria. Furthermore, the capacity of the bifidobacterial strain to compete with, and displace, enteropathogens, using an enterocyte adhesion assay, was also tested.

## 2. Materials and Methods

### 2.1. Bacterial Strains and Growth Conditions

*B. infantis* IM-1^®^, from Laboratorios Ordesa, and *Bifidobacterium animalis* subsp. *lactis* Bb12 were used in the present work. The enteropathogens *Escherichia coli* LMG2092, *Listeria monocytogenes* LMG13305, *Cronobacter sakazakii* LMG5740, *Clostridium difficile* LMG21717, *Salmonella enterica* subsp. *enterica* LMG15860, *Yersinia enterocolitica* LMG7889 and *Shigella sonnei* LMG10473 were obtained from the Belgium Coordinated Collection of Microorganisms (BCCM^TM^; University of Ghent, Belgium). Bifidobacteria were routinely cultured in Man, Rogosa and Sharpe broth (MRS; Merck, Darmstad, Gemany) supplemented with a 0.05% of L-cysteine (Sigma Chemical, St Louis, MO, USA) (MRSc), at 37 °C in anaerobic conditions (10% H_2_, 10% CO_2_ and 80% N_2_) using a chamber Mac 500 (Don Whitley Scientific, West Yorkshire, UK). For bifidobacteria/enteropathogen co-culture experiments, all the pathogens were first grown in brain heart infusion broth (BHI; Merck) at 37 °C in anaerobic conditions. For the adhesion to colonocytes, all the pathogens were grown in Gifu anaerobic medium (GAM; Nissui, Japan) to obtain the cultures for the adhesion experiments. Overall, in order to obtain a standardized culture for the various experiments, the corresponding strains were first streaked onto MRSc (*Bifidobacterium*) or BHI/GAM (*E. coli, L. monocytogenes, Cr. sakazakii, C. difficile, S. enterica, Y. enterocolitica, Sh. sonnei*) agar plates which were incubated anaerobically at 37 °C for 1–2 days. Then, a single colony was inoculated to either MRSc or BHI/GAM and grown overnight, under the same conditions. Before the utilization of these cultures for the different assays described below, cells were washed in sterile Ringer solution to prevent the carry-over of residual carbon sources from the overnight media.

### 2.2. Carbon Source Preferences of the Strain IM-1^®^

#### 2.2.1. Growth Assays

To test the effects of the prebiotics on growth, several culture media, a wide range of bacterial inoculums and two different methodology approaches were chosen. As a basal medium, Man, Rogosa and Sharpe broth without any carbon source added (MRSF) was supplemented with 0.05% of L-cysteine hydrochloride monohydrate (MRSFc). In addition, two commercial infant formulae in powder, Blemil Plus 1 (LAC(+)) and Blemil Plus SL (LAC(-), lacking lactose) from Ordesa laboratory, were employed. The composition of LAC(+) and LAC(-) formulae are presented in Supplementary Table S1. To each of these media, FOS, FOS:Inulin mixture (50:50) and arabinogalactan were added at a final concentration of 0.8%. MRSFc basal medium and both commercial infant formulae without prebiotic supplementation were used as controls in these experiments. An overnight culture of the probiotic strain was prepared by inoculating MRSc as previously described. Before subculturing cells from this overnight into the three media supplemented with different prebiotics, cells were washed and resuspended in a Ringer solution and then added to the culture medium to a final concentration ranging from 10^3^ to10^6^ CFU/mL, in order to evaluate the effect of the inoculum dose. Samples were taken at 8, 24, 32 and 48 h of incubation to determine the optical density (OD) at 600 nm and/or microbial counts. Microbial counts were determined by performing tenfold serial dilutions in Ringer solution, and spreading them into MRSc agar plates which were incubated 2–3 days anaerobically at 37 °C.

#### 2.2.2. Determination of the Best Prebiotic Oligosaccharide Mixture

Based on preliminary studies, MRSFc (10 mL) was used as a basal medium to examine the growth of *B. infantis* IM-1^®^ in the absence or presence of an expanded array of prebiotic carbohydrates. The working oligosaccharide mixtures were FOS, FOS:inulin (50:50), FOS:inulin (75:25), FOS:frutalose (50:50), GOS, GOS:FOS (96:04), GOS:FOS:inulin (96:02:02), GOS:frutalose (96:04), frutalose, and frutalose:FOS:inulin (50:25:25). In accordance with previous results, the optimal bacterial inoculum, 10^6^ CFU/mL, was used. Cultures were incubated under controlled anaerobic conditions at 37 °C for a period of 24 h. Growth of the probiotic strain was monitored by measuring the optical density (OD_660 nm_) at 2 h time intervals.

### 2.3. Inhibition of Pathogen Growth

MRSFc medium (50 mL) supplemented with the appropriate prebiotic (0.8%), selected based on the results of the test described above, was pre-reduced overnight before utilization and a cell suspension, previously washed with a sterile Ringer solution, of both probiotic and pathogenic (ratio 1:1) strains was inoculated at a final concentration of 10^6^ CFU/mL. In addition, pathogen and bifidobacteria single cultures were conducted in parallel in the same media as co-cultures. Both co-cultures and mono-cultures were incubated anaerobically at 37 °C for 24 h. Then, tenfold dilutions of the mono and co-cultures were prepared with Ringer solution and were spread in duplicate on the following selective media: MRSc (Merck) adjusted to pH 5.4 for *Bifidobacterium*, clostridium difficile agar (CLO; bioMériux, Marcy l’Etoile, France) for *C. difficile*, chromogenic listeria agar base (CLAB; Oxoid Ltd., Hampshire, UK) for *L. monocytogens* and violet red bile glucose agar (VRBGA; Oxoid) for *E. coli* and *Cr. sakazakii*. Plates were incubated for 1–2 days at 37 ºC. As an additional effort to improve the selectivity of the media, plates used for pathogen enumerations were aerobically incubated, with the exception of *C. difficile*, to avoid the growth of bifidobacteria, especially in those samples obtained from co-cultures. Cultures were performed in triplicate for each probiotic, pathogen, or probiotic–pathogen combination.

To assay the evolution of the inhibitory activity during 24 h cultivation, new experiments were performed for those combinations that exhibited anti-pathogenic activity. One milliliter of fermentation MRSFc broth was removed at 0, 4, 8, 12, and 24 h, and was serially tenfold diluted in Ringer solution and spread on appropriate selective agar culture media to monitor the growth and inhibition of the probiotic and pathogenic strains, respectively.

### 2.4. Pathogen Displacement and Prevention of Pathogen’s Adhesion to Enterocytes

HT29 monolayers were used in the experiments described next, and prepared as previously described [34]. For pathogen displacement, overnight cultures of the enteropathogen strains (*C. difficile*, *Cr. sakazakii*, *Y. enterocolitica*, *Sh. sonnei*, *S. enterica* and *E. coli*) obtained as previously described, were washed twice in sterile phosphate buffered saline (PBS) solution and bacterial cells were resuspended in supplemented McCoy’s medium (10% foetal bovine serum, 3 mM L-glutamine) (MM; Sigma) without antibiotics, in a ratio of 1:10 (bacteria/enterocyte) prior to its addition to the enterocyte monolayer. The mixture was incubated for 1 h at 37 °C/5% CO_2_ and subsequently the monolayer was washed twice with PBS in order to remove the non-adhered pathogens. Then, an overnight culture of *B. infantis* IM-1^®^ was washed twice with PBS, added to the monolayer (1:10 ratio) and incubated for 1 h, ending with a final washing step to remove unbound bacteria. Afterwards, the monolayers were trypsinized in order to release the HT29 cells and counts of the adhered bacteria were carried out by performing serial tenfold dilutions in Ringer solution and spreading in CLO plates for *C. difficile* and in VRBGA plates for the rest of the pathogen strains (*Cr. sakazakii, Y. enterocolitica, Sh. sonnei, S. enterica* and *E. coli*). All plates, except for those used for *C. difficile* counts, were incubated aerobically at 37 °C. Results were expressed as the percentage of bacteria adhered with respect to the bacteria added (% CFU adhered/CFU added). For comparison purposes, adhesion of the enteropathogens in the absence of bifidobacteria was used as reference for data normalization (% pathogen adhesion in the presence of bifidobacteria/pathogen adhesion in the absence of bifidobacteria).

To evaluate the prevention of the adhesion of the enteropathogen by the strain *B. infantis* IM-1^®^, a different experimental setup was performed. In this case, the enterocyte monolayer was first treated with the strain *B. infantis* IM-1^®^. After 1 h of incubation, non-adhered bifidobacteria were removed and the pathogen was added, incubating the co-culture for 1 h. Following trypsinization, plate counts were carried out for the pathogens by performing serial tenfold dilutions in Ringer solution and using the same selective media and incubation conditions as describe for pathogen displacement assays. Bacteria:enterocyte ratios, bacterial growth conditions and washing procedures were the same as those used for displacement assays.

Two different biological replicates (two independent cultures for each pathogen and probiotic and two independent technical replicates for each culture) were performed. The widely used probiotic *B. animalis* subsp. *lactis* Bb12 was included in the adhesion assays for comparison purposes.

### 2.5. Statistical Analysis

Statistical analysis of the data was performed using the 3.2.5. version of the free R software (The R Foundation, Boston, MA, USA). The differences between single and co-cultures for prebiotic preference and pathogen inhibition data were assessed using Student’s t-test. In adhesion assays, differences in pathogen adhesion among non-bifidobacterial treatment, treatment with *B. animalis* subsp. *lactis* Bb12 or treatment with *B. infantis* IM-1^®^ were assessed using ANOVA tests followed by Tukey’s pairwise mean comparison.

## 3. Results and Discussion

### 3.1. Selection of Prebiotic Candidates

**Figure 1:**
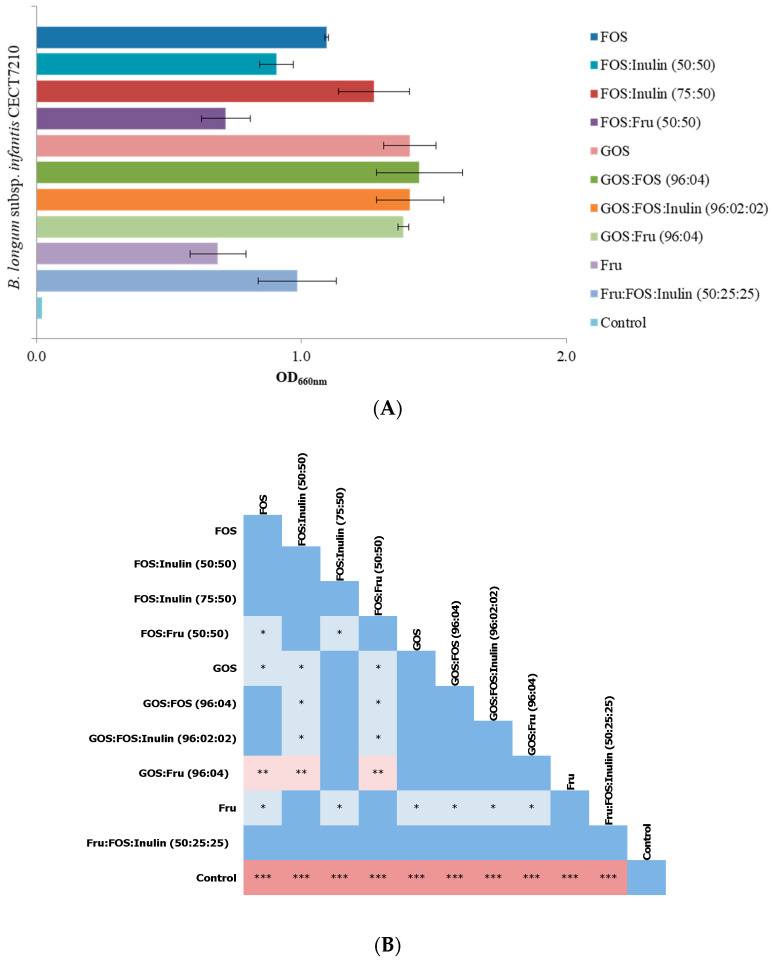
Effect of prebiotics on the growth of *B. infantis* IM-1^®^ after 24 h of incubation. (**A**) Optical density (OD_660 nm_) of the cultures of the *B. infantis* IM-1^®^ strain grown in the presence of different prebiotic substrates. The represented data are means of at least three independent replicates. (**B**) Statistically significant differences in growth of *B. infantis* IM-1^®^ in the presence of different prebiotics were determined, using Student’s *t*-test for each pair of substrates tested. Color key: dark blue: *p* > 0.05; light blue: * *p* < 0.05; light red: ** *p* < 0.01; and dark red: *** *p*. < 0.001. FOS, fructooligosaccharides; GOS, galactooligosaccharides; Fru; Frutalose, and control (MRSFc: Man, Rogosa and Sharpe broth with 0.05% of L-cysteine hydrochloride monohydrate. MRSFc without prebiotic supplementation).

In a preliminary assay, we defined the optimal basal media formulation, dose of bacterial inoculum, and incubation period to achieve consistent and reproducible growth of *B. infantis* IM-1^®^, as well as to detect differences in the growth that could be attributed to the fermentation of the different prebiotic oligosaccharides used. For this purpose, prebiotic oligosaccharides (FOS, FOS:Inulin mixture and arabinogalactan) were tested in different media, including MRSFc (nutrient-limited medium) and two infant formulae: one containing lactose (LAC(+)) and one lacking lactose (LAC(−)) (nutrient-rich media). Using the plate count technique, we observed that *B. infantis* IM-1^®^ grew well in basal medium (MRSFc), and in both infant formulae (LAC(+) and LAC(−)) supplemented with prebiotics. The values of mean log counts were 7.68 ± 0.61 log CFU/mL for MRSFc, 7.96 ± 0.11 log CFU/mL for LAC(+) and 7.40 ± 0.43 log CFU/mL for LAC(−). However, no significant differences in the number of viable cells were observed after 48 h of incubation between all cultures media supplemented with prebiotics and controls (media without prebiotics) (7.71 ± 0.75 log CFU/mL with prebiotics vs. 7.91 ± 0.32 log CFU/mL without prebiotics). Unlike this fact, a great variation on prebiotic effects was detected in the OD levels of *B. infantis* IM-1^®^ measured in basal media (MRSFc), mainly with FOS and FOS:inulin in the medium, obtaining values ranging from 35 to 56 fold higher than in the non-supplemented MRSFc control medium. The highest differences between the presence or absence of prebiotics were observed at the highest bacterial inoculum concentration tested. Specifically, OD values obtained compared to no prebiotic added, were from 16- to 17-fold higher for arabinogalactan; from 35 to 46 for FOS:Inulin; and from 42 to 56 for FOS, when bacterial inoculum doses were 10^5^ or 10^6^ CFU/mL, respectively. Using lower doses of bacterial inoculum (10^3^ and 10^4^ CFU/mL), growth in MRSFc was not observed either in the absence or presence of prebiotics. In addition, incubations longer than 24 h did not result in an increase in growth of *B. infantis* IM-1^®^. In view of these results, we selected MRSFc, a bacterial dose of 10^6^ CFU/mL, and OD600 nm after a 24 h incubation period to monitor bacterial growth in subsequent batch fermentation assays with a variety of prebiotic combinations. In this respect, the highest OD occurred with GOS and all combinations including this oligosaccharide: GOS:FOS, GOS:FOS:inulin and GOS: frutalose (Figure 1). OD of *Bifidobacterium* cultures also increased in the presence of FOS or FOS:inulin combinations, whereas the lowest growth was achieved in the prebiotic frutalose as a carbon source.

The ability of FOS and GOS, and the mixture of both of them, to enhance the growth of bifidobacteria populations of the colonic microbiota have been previously reported [18,35,36,37]. Several reports showed the beneficial effect of prebiotic carbohydrates on the growth of probiotic strains. However, a great variability in the response of different probiotic strains to different prebiotic carbohydrates exists, suggesting that there is not a universal prebiotic to design synbiotic formulations and highlighting the fact that the ability of probiotic strains to grow in synbiotic combinations with different prebiotics may be strain-specific and must be determined independently.

### 3.2. B. infantis IM-1^®^ Inhibits Growth of Enteropathogens In Vitro

**Figure 2:**
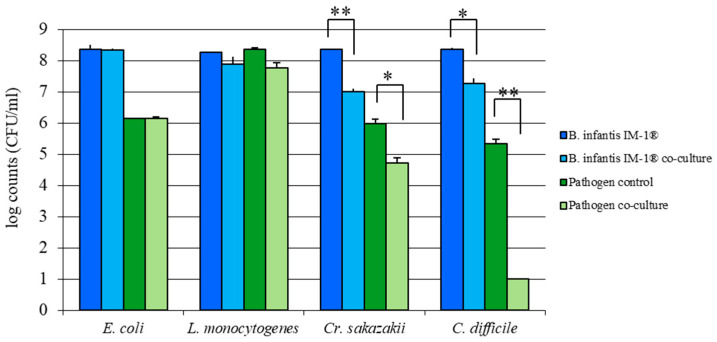
Mean log counts (CFU/mL) and standard deviations of enteropathogenic strains (*E. coli* LMG 2092, *L. monocytogenes* LMG 13305, *Cr. sakazakii* LMG 5740 and *C. difficile* LMG 21717; pathogen co-culture) and *B. infantis IM-1^®^* strain (*B. infantis IM-1^®^* co-culture) after 24 h of co-culture in the presence of GOS. Single cultures of each bacteria type were also performed using the same media and incubation conditions (*B. infantis* IM-1^®^ and pathogen control). For each pathogen–bifidobacteria combination, the statistical significance of counts between co-culture and single culture (control) was calculated using Student’s t-test (* *p* < 0.001, ** *p* < 0.0001).

The antimicrobial activity of the strain *B. infantis* IM-1^®^ against several intestinal pathogens was evaluated *in vitro* with co-culture assays, and data were collected after 24 h of incubation. *B. infantis* IM-1^®^ was inoculated simultaneously with the enteropathogens in MRSFc supplemented with GOS (0.8% final concentration). After 24 h, the growth of pathogens and *B. infantis* IM-1^®^ was determined by plating cultures on selective media and colony counting (see Methods). In co-cultures, the same sample was plated onto selective medium for probiotic strain (MRSc pH 5.4) and also onto selective medium for pathogen (CLO, CLAB or VRBGA depending on pathogen) (see Methods). Under these conditions, the greatest inhibition was observed in the growth of *C. difficile* during co-culture, so the decrease was of 4–5 log values with respect to the pathogen control culture (pathogenic strain grown in the absence of the bifidobacteria in the same MRSFc supplemented with GOS medium and incubated under identical conditions) (Figure 2). Furthermore, *Cr. sakazakii* growth was significantly inhibited by 1–2 log values during co-culture with *B. infantis* IM-1^®^, compared to single pathogen cultures. By contrast, no significant inhibition of *E. coli* or *L. monocytogenes* was exerted by *B. infantis* IM-1^®^, with identical microbial counts of these bacteria recovered when grown in mono- and co-cultures.

*B. infantis* IM-1^®^ strain has been demonstrated to prevent diarrhea episodes in formula-fed infants and to provide protection against rotavirus infection in various experimental models [30,31,32]. However, its capacity to antagonize other enteropathogens of relevance for infant health had not been explored. Our results demonstrate that *B. infantis* IM-1^®^ strain is capable of reducing the growth of various enteropathogens *in vitro*, as previously reported for other bifidobacterial species/strains. For example, isolates belonging to the genera *Bifidobacterium* have been employed in the treatment of gastrointestinal diseases caused by *C. difficile* [38,39]. In addition, it has been previously reported that the increase in bifidobacteria populations in the presence of FOS and GOS promoted the inhibition of *C. difficile* in vitro [40]. Similarly, co-cultures of bifidobacteria with short chain FOS inhibited *C. difficile* growth [41]. On the other hand, our results are in contrast with the antipathogenic activity against *E. coli* observed in other species of *Bifidobacterium* [42,43]. Indeed, several studies have reported the existence of high variability in the antipathogenic activities exerted by different strains belonging to the same genus/species, further supporting the idea that health-promoting and probiotic traits are strain-specific and need to be evaluated individually for every probiotic candidate [44,45,46].

**Figure 3:**
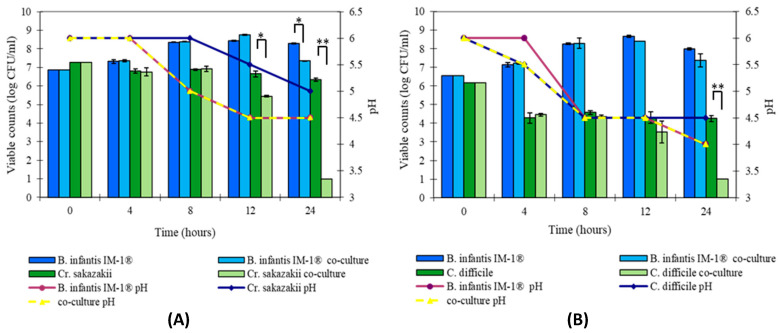
Evolution of microbial counts (log CFU/mL) and pH values in single and co-cultures of probiotic strain *B. infantis* IM-1^®^ and enteropathogens: (**A**) *Cr. sakazakii* and (**B**) *C. difficile*. For each pathogen–bifidobacteria combination, the statistical significance of counts between co-culture and single culture (control) was calculated using Student’s *t*-test (* *p* < 0.001, ** *p* < 0.0001).

To go deeper into the inhibition activity capability of *B. infantis* IM-1^®^ against *C. difficile* and *Cr. sakazakii*, new assays of co-culture were performed checking the growth decrease over time. Figure 3 represents the microbial counts (log CFU/mL) of the probiotic and pathogenic bacteria in single and co-cultures, as well as pH variations. The effect of antibacterial activity of strain *B. infantis* IM-1^®^ against *C. difficile* growth during co-culture appears after 8 h, and no viable cells were detected after 24 h. Meanwhile, *Cr. sakazakii* growth showed a moderate decrease after 8–12 h in co-culture with *B. infantis* IM-1^®^, and a much greater decrease after 24 h.

Similar results have been previously described [47] in *Lactobacillus acidophilus* LB against *Salmonella typhimurium* SL1344, where antipathogenic activity began after 12 h in culture. This is consistent with some reports that have demonstrated *C. difficile* inhibition by culture supernatants of *B. longum* and *Bifidobacterium breve* strains, an effect that was dependent on the prebiotic substrates used to grow bifidobacteria [41]. Indeed, some works have indicated that *Bifidobacterium* strains can be used in the treatment of *Clostridium*-associated diarrhea [48,49]. The selection of *Bifidobacterium* strains able to inhibit clostridia growth is therefore important for the development of probiotic products targeted to prevent gut colonization by *C. difficile*. However, the specific mechanisms responsible for the pathogen growth inhibition observed in pathogen co-cultures with *Bifidobacterium* strains remain to be elucidated. A possible effect of pH decrease or the production of antimicrobial substances active against pathogenic bacteria, as demonstrated for other species/strains [50], cannot be ruled out and deserves further attention.

### 3.3. Capability of Probiotic Strain B. Infantis IM-1^®^ to Modify the Adhesion of Enteropathogens to HT29

**Figure 4:**
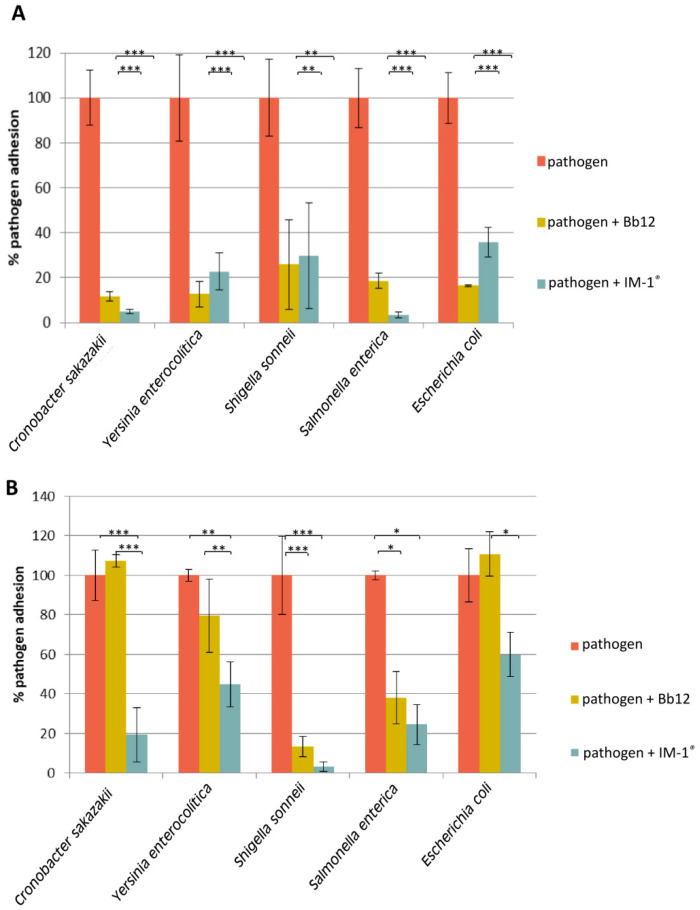
Retained pathogen adhered to HT29 cells after subsequent displacement by exposition to *B. infantis* IM-1^®^ cells (**A**); and pathogen adhesion to HT29 cells previously exposed to *B. infantis* IM-1^®^ cells (**B**). The results were normalized considering 100% the adhesion of the pathogen in the absence of bifidobacterial treatment. The strain *B. animalis* subsp. *lactis* Bb12 was used as a reference for comparative purposes. For each pathogen, the three treatment groups were compared to get statistical significance by using ANOVA tests, followed by Tukey pairwise test comparison (*** *p* < 0.001, ** *p* < 0.01, * *p* < 0.05).

In order to determine the ability of *B. infantis* IM-1^®^ to displace or to prevent the adhesion of several enteropathogens to the intestinal epithelium, adhesion assays using the enterocyte cell line HT29 were carried out. In addition, *B. animalis* subsp. *lactis* Bb12 strain was used as a probiotic reference due to the fact that it is a model probiotic strain widely used in commercial probiotic products, known to exhibit relatively high adhesion in vitro to intestinal cell models, such as the one used in the present work, and for which the capability to prevent certain pathogens adhesion has already been documented [51]. Unfortunately, *C. difficile* was not able to survive after the microaerophilic conditions used during the adhesion assay, and viable cells could not be recovered, thus preventing the evaluation of the strain *B. infantis* IM-1^®^ capacity to prevent or displace this pathogen adhesion. The strain *B. infantis* IM-1^®^ was able to displace all the other pathogenic strains tested (Figure 4A), especially *Cr. sakazakii* and *S. enterica* in a similar way to the strain *B. animalis* subsp. *lactis* Bb12. Furthermore, the previous adhesion of *B. infantis* IM-1^®^ decreased the adhesion of all the pathogens to HT29 cells, although the effect was more pronounced for *Sh. sonnei* and *Cr. sakazakii* (Figure 4B). In general, *B. infantis* IM-1^®^ was more effective than the reference probiotic (strain Bb12), except for *Sh. sonnei* and *S. enterica*, for which prevention of adhesion in the presence of both probiotics was similar (no statistical differences, Figure 4B).

Several reports have studied the effect of probiotic strains on pathogen adhesion using intestinal cell models. In this regard, different scenarios could be considered: probiotics can directly compete for adhesion sites in the intestine, or displace the already adhered pathogens, or even prevent the attachment of the pathogens when adhesion sites are blocked by the probiotic strain. *Bifidobacterium* strains have been able to act on these three possible scenarios [24,25,52,53,54,55,56]. However, the array of pathogens whose adhesion might be reduced by probiotic bifidobacteria, seems to be strain-dependent. In this regards, the strain *B. infantis* IM-1^®^ seems to display a preferential activity on *Cr. sakazakii*, its inhibition effect being better than for the strain *B. animalis* Bb12 that was used as a control. This suggests a promising effect of the strain *B. infantis* IM-1^®^ to act on *Cr. sakazakii*-induced infections, which are especially relevant in infants [57].

## 4. Conclusions

This study confirmed that the addition of a single oligosaccharide, or a mixture of oligosaccharides, has positive effects on the growth promotion of *B. infantis* IM-1^®^, a bifidobacterial strain originally isolated from the feces of a breastfed infant. Based on the results of this work, we propose that suitable synbiotic combinations including the *B. infantis* IM-1^®^ probiotic strain should contain either GOS or GOS-containing mixtures. In addition, co-culture experiments confirmed the ability of *B. infantis* IM-1^®^ grown in the presence of GOS to inhibit *C. difficile* and *Cr. sakazakii* when grown in co-culture. This probiotic strain was also able to prevent adhesion, and to efficiently displace some pathogens, especially *Cr. sakazakii*. Therefore, this work expands on the antipathogenic potential of bifidobacterial strains isolated from particular population groups (breast-fed infants) and provides the rationale to design novel functional foods and synbiotic combinations including the *B. infantis* IM-1^®^ strain. Therefore, we propose that the *B. infantis* IM-1^®^ strain, which had already been demonstrated to reduce diarrhea episodes in infants and to prevent rotavirus infection, also has the potential to antagonize a range of other enteropathogens *in vitro*. For these reasons, the results of this work support its potential benefits as a probiotic strain in infant foods specifically designed for formula-fed infants and, particularly, when formulated in synbiotic combinations including GOS or GOS-containing oligosaccharides. Further studies are needed to complete the characterization of the anti-pathogenic activity of this strain and to elucidate its mechanisms of action.

## Supplementary Materials

The following are available online at https://www.mdpi.com/2072-6643/12/11/3259/s1, Table S1: Nutritional composition of the two infant formulas used in the study.
